# A Promising Tool to Achieve Chemical Accuracy for Density Functional Theory Calculations on Y-NO Homolysis Bond Dissociation Energies

**DOI:** 10.3390/ijms13078051

**Published:** 2012-06-28

**Authors:** Hong Zhi Li, Li Hong Hu, Wei Tao, Ting Gao, Hui Li, Ying Hua Lu, Zhong Min Su

**Affiliations:** 1 School of Computer Science and Information Technology, Northeast Normal University, Changchun 130017, China; E-Mails: lihz857@nenu.edu.cn (H.Z.L.); gaot080@nenu.edu.cn (T.G.); lihui@nenu.edu.cn (H.L.); 2 School of Life Sciences, Northeast Normal University, Changchun 130024, China; 3 Institute of Functional Material Chemistry, Faculty of Chemistry, Northeast Normal University, Changchun 130024, China; E-Mail: taow587@nenu.edu.cn

**Keywords:** Y-NO bond, homolysis bond dissociation energies, density functional theory, self-organizing feature mapping neural network, radial basis function neural network

## Abstract

A DFT-SOFM-RBFNN method is proposed to improve the accuracy of DFT calculations on Y-NO (Y = C, N, O, S) homolysis bond dissociation energies (BDE) by combining density functional theory (DFT) and artificial intelligence/machine learning methods, which consist of self-organizing feature mapping neural networks (SOFMNN) and radial basis function neural networks (RBFNN). A descriptor refinement step including SOFMNN clustering analysis and correlation analysis is implemented. The SOFMNN clustering analysis is applied to classify descriptors, and the representative descriptors in the groups are selected as neural network inputs according to their closeness to the experimental values through correlation analysis. Redundant descriptors and intuitively biased choices of descriptors can be avoided by this newly introduced step. Using RBFNN calculation with the selected descriptors, chemical accuracy (≤1 kcal·mol^−1^) is achieved for all 92 calculated organic Y-NO homolysis BDE calculated by DFT-B3LYP, and the mean absolute deviations (MADs) of the B3LYP/6-31G(d) and B3LYP/STO-3G methods are reduced from 4.45 and 10.53 kcal·mol^−1^ to 0.15 and 0.18 kcal·mol^−1^, respectively. The improved results for the minimal basis set STO-3G reach the same accuracy as those of 6-31G(d), and thus B3LYP calculation with the minimal basis set is recommended to be used for minimizing the computational cost and to expand the applications to large molecular systems. Further extrapolation tests are performed with six molecules (two containing Si-NO bonds and two containing fluorine), and the accuracy of the tests was within 1 kcal·mol^−1^. This study shows that DFT-SOFM-RBFNN is an efficient and highly accurate method for Y-NO homolysis BDE. The method may be used as a tool to design new NO carrier molecules.

## 1. Introduction

Over the past two decades, first-principles calculations have become an attractive complement or alternative to wet chemistry experiments for studying molecular properties and chemical reaction mechanisms. Great progress has been made: calculation speed has accelerated and the size of the target molecules has increased, as has the computational accuracy [1–3]. The applications of first-principles methods are rather extensive. In some studies, they have already gone beyond the level of testing and verifying experiments to predicting the properties of molecules under experimental circumstances that have not undergone real-life tests [4–8]. However, current first-principles calculations cannot yet meet the high accuracy needed for databases with large numbers of medium or large molecules. Deviations in calculations arise from various sources: some from inherent programs, approximations and simplifications in formulas and some from the choice of software, methods, basis sets, and so forth. In addition, we have to admit that each molecule is unique, but computational program cannot fully cover the uniqueness of each molecule, some deviations induced by unified calculations are unavoidable. These deviations can be corrected to improve calculations. Computational theory can be improved, for example, by modifying functions, avoiding approximations, and using an infinite basis set. However, these corrections are time-consuming, and the effect might be insignificant. An alternative is to correct calculation results through statistical methods, which may improve the calculations significantly in a simple and fast way and simplify the prediction of new compounds [9–18]. The method is quite useful for improving functional molecule design and can guide synthetic chemists in choosing potential target compounds. In particular, machine learning methods have recently become a new option to solve wave function problems [12,19].

One first-principles method, hybrid density functional theory (DFT) has become very popular in recent years because of its efficiency and accuracy. With the introduction of exchange and correlation functionals, DFT costs much less than other high-level *ab initio* methods (such as MP2 and CI), and its accuracy can be as good as those methods. Nevertheless, DFT calculations need further improvement to achieve highly accurate results, especially for medium or large molecules [7–9]. The DFT-NEURON method from the Chen group combines neural networks and DFT methods, setting up a quantitative relationship between experimental values and DFT calculation results using a neural network to improve DFT calculation accuracy. The first application of this method was made to the heats of formation for 180 organic molecules; the root mean square errors were reduced from 21.4 kcal·mol^−1^ to 3.3 kcal·mol^−1^ for B3LYP/6-311 + G(d,p) calculations [9]. In the study, the neural network or machine learning method showed its substantial potential to improve the efficiency of first-principles calculations. The method has since been successfully applied to other properties [10–18], and the concept is applicable to other quantum chemical methods.

However, only a few reports investigate preprocessing molecular descriptors [14,15] (the inputs of a neural network). These inputs are crucial for calculations because they greatly influence the capability of the network. Molecular descriptors can be obtained from the structure or properties of systems and can be diverse, including constitutional, topological, electrostatic, geometrical, and quantum chemical descriptors [20]. Without a selection procedure, molecular descriptors are usually selected subjectively according to the knowledge and experience of researchers, who may overlook very important information related to the quantity of interest or inadvertently overlay this information with noise. Chemists may think some descriptors are trivial when they are actually critical for the statistical calculations. With hundreds of molecular descriptors, it is difficult to make prudent choices relying only on intuition and experience. Therefore, in this study, we introduce SOFMNN clustering analysis and correlation analysis to refine molecular descriptors as the inputs of neural networks.

Nitric oxide (NO) performs significant physiological functions in human life processes [21–30]. The highly active free radical NO must be carried by a linear molecular precursor, so NO homolysis (formation/breaking of the bond between NO and the rest of the molecule) BDE is of interest for the medicinal study of NO-release diseases. Because the experiments are complicated, homolysis BDE of NO carrier molecules is difficult to measure with high accuracy. Recently, the Cheng group has focused much effort on measurements of homolysis BDE of the Y-NO bond in solution [31–41], which has greatly contributed to NO molecular carrier design *in silico*.

In this article DFT, SOFMNN and RBFNN methods are combined to improve the accuracy of the calculations of homolysis Y-NO BDE by DFT. The first section describes the neural network methods SOFMNN and RBFNN; the second section describes calculations using the DFT B3LYP method with two basis sets, 6-31G(d) and STO-3G, and the collection of the calculated homolysis BDE and relevant molecular descriptors of Y-NO bond; the third part discusses the calculation results from the DFT, SOFMNN and RBFNN methods, as well as classifying appropriate molecular descriptors by the SOFMNN method, setting up RBFNN and optimizing the non-linear model for both B3LYP results. In the last section, our conclusions are summarized.

## 2. Methods

### 2.1. Self-Organizing Feature Mapping Neural Network

**Figure 1:**
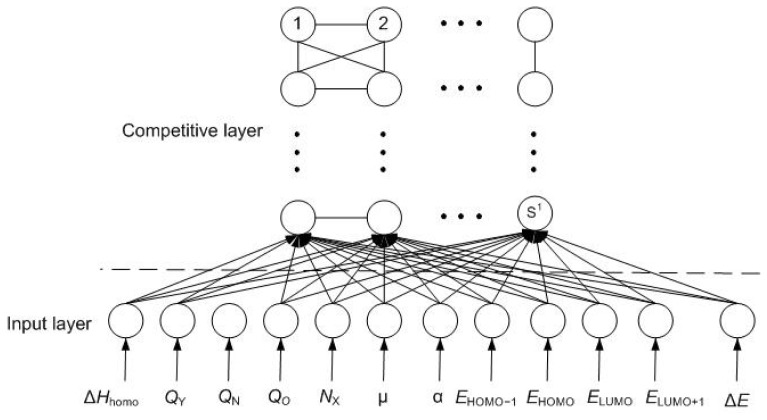
The structure of self-organizing feature mapping neural network (SOFMNN).

Self-organizing feature mapping neural network (SOFMNN) was proposed by Kohonen in 1981 around the concept that an ordered arrangement of neurons could reflect certain physical properties of sensed external stimuli [42]. The main idea is to gradually reduce the interaction areas of neurons in the study process and strengthen the activation of central neurons per relevant learning rules, allowing the removal of neural connections to achieve a model of the real brain nervous system that “excited the nearby neurons while retaining the far-away ones.” The structure of SOFMNN consists of input layers and competitive layers (aka mapping layers) (shown in Figure 1).

A characteristic of SOFMNN is that the featured topology distribution of the input signal can be established in terms of an array of one-dimensional or two-dimensional processing units so that SOFMNN may extract features of the input signal. This is of great importance to correct first-principles calculations using the neural network because the neural network must extract precisely the essential information from inputs obtained by first-principles methods. Calculations over the past few decades have proved that primarily first-principles methods can capture the physical essence of molecules. These characteristics of SOFMNN are the strength of our DFT-SOFM-RBFNN method to achieve high-accuracy calculations. The procedures of the SOFMNN learning algorithm are as follows:

1. Network initialization
  The input layer and competitive layer are composed of *R* and *S*^1^ neurons, respectively. The initial values of each neuron in the competitive layer start from a small random number *IW_ij_*^1,1^ (*i* = 1,2,…, *S*^1^, *j* = 1,2,…,*R*), where *IW_ij_*^1,1^ represents the connection weight between the *i^th^* neuron in the competitive layer and the *j^th^* neuron in the input layer. *N_c_* is set as the initial neighborhood, η as the initial learning rate, *T* as the maximum iterations, and *N* = 1 as the initial iteration.
2. The winning neuron calculation
  A training sample *p* is selected randomly and the input of neurons in the competitive layer is calculated according to Equation (1)
  (1) $$n_{i}^{1}=-\sqrt{\sum_{j=1}^{R}\left(p^{j}-IW_{ij}^{1,1}\right)}+b_{i}^{1},i=1,2,\cdots ,S^{1}$$
  where *n_i_^1^* and *b_i_^1^* represent the output and the threshold value of the *i^th^* neuron in the competitive layer, respectively; *p^j^* stands for the value of the *j^th^* input variable of sample *p*.
  If the *k^th^* neuron in the competitive layer is the winning neuron, it should meet the requirements in Equation (2):
  (2) $$n_{k}^{1}=\text{max}\left(n_{i}^{1}\right),i=1,2,\cdots S^{1},k\in \left\lfloor 1,S^{1}\right\rfloor$$
3. Weight update
  The weights of the winning neuron *k* and all neurons in neighborhood *N_c_*(*t*) will be updated according to Equation (3):
  (3) $$\{\begin{array}{l} IW_{j}^{1,1}=IW_{j}^{1,1}+\eta (t)\left(p-IW_{j}^{1,1}\right),j\in N_{c}(t)\\ IW_{j}^{1,1}=IW_{j}^{1,1},j\notin N_{c}(t) \end{array}$$
4. Learning rate and neighborhood neurons update
  Once the weights of the winning neuron and the neighborhood neurons are updated, the learning rate and neighborhood neurons must be updated before the next iteration according to Equations 4,5:
  (4) $$\eta =\eta \left(1-\frac{N}{T}\right)$$
  (5) $$N_{c}=\left\lceil N_{c}\left(1-\frac{N}{T}\right)\right\rceil$$
  where the operator ⌈ ⌉ represents rounding up.
5. Iteration
  If the learning process is not finished, another sample will be randomly chosen to continue the calculation, and the iteration returns to step (2), or if *N* < *T*, then *N* = *N* + 1, and iteration also returns to step (2). Otherwise, iteration concludes.

### 2.2. Radial Basis Function Neural Network

**Figure 2:**
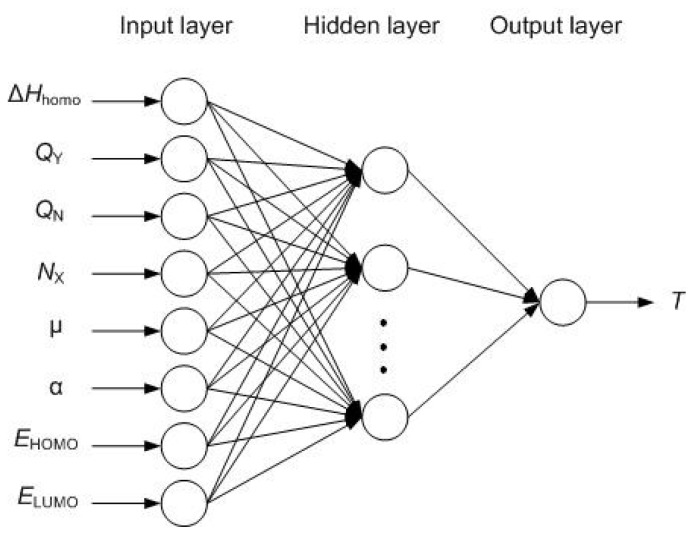
The structure of radial basis function neural network (RBFNN).

In 1985, Powell proposed the radial basis function (RBF) method of multivariable interpolation [43]. In 1988, Moody and Darken came up with a neural network structure, *i.e.*, RBFNN, which can approach any continuous function with various accuracies. RBFNN is a three-layered feed-forward network. The network structure is shown in Figure 2.

The basic idea of RBFNN uses RBF as the “basis” of neurons in the hidden layer to construct the hidden layer space. Thus, input vectors can be mapped directly to the hidden layer space without weights between the input layer and hidden layer. Once the RBF central point is determined, the mapping relationship is determined. The mapping from the hidden layer space to the output layer space is linear, *i.e.*, the output is the sum of linear weighted neurons in the hidden layer, where the weight is the adjustable parameter of the network. Generally, network mapping from input to output is non-linear, while the output is linear to adjustable parameters. In this way, the weight of the neural network can be solved directly from linear equations so that learning rate will improve significantly and local minimum problems will be avoided.

The specific steps of the learning algorithm of the RBFNN are as follows:

1. Determining the RBF center of neurons in the hidden layer
  The input matrix *P* and output matrix *T* for the training set can be described in Equation (6):
  (6) $$P=\left[\begin{array}{cccc} t_{11} & t_{12} & \cdots & t_{1Q}\\ t_{21} & t_{22} & \cdots & t_{2Q}\\ \vdots & \vdots & & \vdots \\ t_{M1} & t_{M2} & \cdots & t_{MQ} \end{array}\right],\;T=\left[\begin{array}{cccc} t_{11} & t_{12} & \cdots & t_{1Q}\\ t_{21} & t_{22} & \cdots & t_{2Q}\\ \vdots & \vdots & & \vdots \\ t_{N1} & t_{N2} & \cdots & t_{NQ} \end{array}\right]$$
  where *p_ij_* represents the *i^th^* input variable of the *j^th^* training sample; *t_ij_* represents the *i^th^* output variable of the *j^th^* training sample; *M* is the dimension of the input variables; *N* is the dimension of the output variables; and *Q* is the number of samples in training set.
  The corresponding RBF center of *Q* neurons in the hidden layer is:
  (7) $$C=P^{\prime }$$
2. Determining the threshold value of neurons in the hidden layer
  The corresponding threshold value of *Q* neurons in the hidden layer is:
  (8) $$b_{1}={\left\lfloor b_{11},b_{12},\cdots ,b_{1Q}\right\rfloor }^{\prime }$$
  where *b*_11_ = *b*_12_ = ··· = *b*_1Q_ = 0.8326/*spread*, spread is the expanding coefficient of RBF.
3. Determining weights and threshold values between the hidden layer and the output layer
  Once the RBF center and threshold value of neurons in the hidden layer is determined, the output of neurons in the hidden layer can be obtained by Equation (9):
  (9) $$a_{i}=\text{exp}(-{\left||C-p_{i}|\right|}^{2}b_{i}),i=1,2,\cdots ,Q$$
  where *p_i_* = [*p_i1_*, *p_i2_*, ···, *p_im_*] is the *i^th^* vector of the training set. And the matrix A is set to *A* = [*a*_1_, *a*_2_, ··· *a*_Q_].

The connection weight *W* between the hidden layer and the output layer is set as Equation 10:

(10) $$W=\left[\begin{array}{cccc} w_{11} & w_{12} & \cdots & w_{1Q}\\ w_{21} & w_{22} & \cdots & w_{2Q}\\ \vdots & \vdots & & \vdots \\ w_{N1} & w_{N2} & \cdots & w_{NQ} \end{array}\right]$$

where *w_ij_* represents the connection weight between the *j^th^* neuron in the hidden layer and the *i^th^* neuron in the output layer.

If the threshold value *b*_2_ of *N* neurons in the output layer is obtained Equation (11):

(11) $$b_{2}={\left[b_{21},b_{22},\cdots ,b_{2N}\right]}^{\prime }$$

The weight *W* and threshold value *b*_2_ between the hidden layer and output layer can be obtained by the linear Equation (12), where *I* = [1,1, ···,1]_1×Q_.

(12) $$[\begin{array}{ll} W & b_{2} \end{array}]\cdot [\begin{array}{lll} A & ; & I \end{array}]=T$$

## 3. Calculations

### 3.1. Data Set

In total, 98 organic molecules were used in the dataset for this study. Six molecules were added to the set of molecules used in our previous study [15] to validate the predictive ability of the neural network. Chemical elements in these molecules include H, C, N, O, F, Si, P, S, Cl and Br, and the number of non-hydrogen atoms in the molecules varies from 8 to 25 for these small or medium molecules. The final RBFNN models are attained according to the relatively stable estimation results of the testing set. Once the neural network is established, the calculations for these data require negligible time to perform, which shows the efficiency of this correction approach.

### 3.2. Molecular Descriptor Calculations

Molecular descriptors should represent typical characteristics of molecules and closely correlate to the quantity of concern. Because we intended to develop an easy-to-use method, simple descriptors were favored. Because the DFT calculation results are corrected and performed for each molecule, quantum chemical descriptors are ready-made. In addition to quantum chemical descriptors, constitutional descriptors such as the molecular weight, number of atoms, and number of electrons are also better descriptors due to their ease of generation. All DFT calculations were performed using the Gaussian03 software package [44]. The DFT calculation for homolysis BDE and twelve molecular descriptors by hybrid functional method B3LYP with 6-31G(d) were described in [15], and the corresponding calculation results by B3LYP/STO-3G method are shown in the Supplementary materials.

## 4. Results and Discussion

### 4.1. Calculating Y-NO Homolysis BDE with DFT Method

The homolysis BDE are calculated using DFT B3LYP method with two basis sets, 6-31G(d) and STO-3G. The minimal basis set STO-3G consists of 1 function for H, 5 functions for Li to F and 9 functions for Na to Cl; the basis set 6-31G(d) consists of 2 functions for H, 15 for Li to F and 19 function for Na to Cl. So for most organic molecules, STO-3G only contains less than half of 6-31G(d) basis functions. Then with the STO-3G basis set much time can be saved during DFT calculations. For example, the B3LYP frequency calculation for molecule 85 takes 114 minutes with the basis set 6-31G(d), while it only takes 13 minutes with the basis set STO-3G. This offers applications for molecules that are quite large. The calculated values of the Y-NO (Y = C, N, O, S) homolysis BDE, the experimental data and the corresponding molecular descriptors of the 92 molecules are listed in Tables 1,2 in the Supplementary materials.

By analyzing the molecular descriptors, we find that, in the B3LYP/6-31G results, the charge on the N atom of NO does not change with the charge on Y. The electronegativity of Y itself is most likely the key factor determining the amount of charge on N because the charge on the N atom only changes with the type of Y atoms. Neither the structure of molecular fragments that connect to Y nor the amount of charges on Y has much effect on the charge value of N. When Y = N, O, S, C, the charges on the N atom of Y-NO are between 0.21–0.25e, 0.38–0.44e, −0.01–0.08e and 0.13–0.17e, respectively; the charges on Y change in the range from −0.63–0.33e, as determined by the rest of molecules. The charges on the O atoms do not fluctuate very much and have no clear pattern. In Table 2 of the Supplementary Materials, the patterns of calculated charges on Y, N and O atoms shown with the STO-3G basis set are consistent with 6-31G(d), although the magnitude of the atomic charges are different. In addition, there are some exceptions for charges on the N atom of N-NO bond (molecules 30–36 and 50–53 have about half charges for the same Y-NO molecules), when N is bonded to S.

Structural analysis indicates that the conformation of the molecules and functional groups on the aromatic rings are shown to affect the homolysis BDE. Conformational effects reported by the Guo group show that syn and anti conformations induce BDE differences between isomers [45]. In our data set, most molecules contain aromatic rings and functional groups that include –CH_3_, –CH_3_O, –Cl, –Br and –NO_2_, among which –CH_3_ and –CH_3_O are electron-donating groups and –Cl, –Br and –NO_2_ are electron-withdrawing groups. The electron-donating groups on the *meta-* and *para*- positions of the benzene ring decrease the BDE of the Y-NO bond, while electron-withdrawing groups on these positions increase the BDE. Electron-donating groups at the *ortho-*position decrease the BDE of the Y-NO bond, but the effects of electron-withdrawing groups are stronger than electron-donating groups. The substitution effects are smaller for molecules with multiple rings (e.g., indole, dibenzo-azepine) than for benzene rings due to the longer distance between the substituent group and the Y-NO bond.

To study the correlation between the molecular descriptors and the Y-NO experimental homolysis BDE, a correlation analysis was performed. The results show that the B3LYP/6-31G(d)-calculated homolysis BDE values (ΔH_homo_) are the most relevant to the experimental homolysis BDE and the correlation coefficient is 0.64, which proves that DFT calculations indeed capture the essence of physics. This is the reason that DFT-calculated homolysis BDE (ΔH_homo_) are considered the primary descriptor. The correlation coefficients of other strong related molecular descriptors are as follows: E_HOMO_(0.51), Q_N_(0.49), Q_Y_(0.46) and E_HOMO-1_(0.43). The remaining descriptors have numerically weaker relationships with the experimental homolysis BDE, and the correlation coefficients are α(0.28), ΔE(0.27), μ(0.18), E_LUMO_(0.17), N_X_(0.12), E_LUMO+1_(0.05) and Q_O_(0.02). For the molecular descriptors calculated by B3LYP/STO-3G, the correlation coefficients in decreasing order are E_HOMO_(0.48), Q_N_(0.43), E_HOMO-1_(0.40), Q_Y_(0.39), ΔH_homo_(0.35), ΔE(0.34), α(0.31), N_X_(0.12), E_LUMO_(0.06), E_LUMO+1_(0.05), Q_O_(0.05) and μ(0.03). The coefficient shows that the calculated ΔH_homo_ by B3LYP/STO-3G has a weaker relationship with the experimental homolysis BDE than that of B3LYP/6-31G(d) due to its poor accuracy. In addition, it can be seen that the types of molecular descriptors strongly related with the experimental homolysis BDE do not change greatly. This suggests that the B3LYP/STO-3G calculation results essentially agree with the B3LYP/6-31G(d) results, but with large deviations.

The deviations of all the methods are listed in Table 1. The total MADs for two basis sets 6-31G(d) and STO-3G are 4.45 and 10.53 kcal·mol^−1^, respectively. For the results of B3LYP/6-31G(d), the deviations between the DFT calculated and experimental homolysis BDE for all four types of carriers span a wide range, from −17.17 to 7.91 kcal·mol^−1^. The calculated homolysis BDE vary according to the type of Y atoms in the Y-NO bond, and the deviation distributions also change with different types of Y-NO bond. The DFT-calculated homolysis BDE of the S-NO bond carrier molecules agree best with the measured values: the MAD is 1.83 kcal·mol^−1^. The DFT calculation results are in particularly good agreement with the experimental data for molecules with amino acid groups (78–84), although the introduced amino acid groups make these molecules the largest in the dataset, and the MAD is only 1.46 kcal·mol^−1^. This may be good news for theoretical studies on the mechanism of physiological release of NO in the human body. The MAD of DFT-calculated homolysis BDE for N-NO bond carrier molecules is 4.75 kcal·mol^−1^, which is much larger than that of the S-NO bond carrier molecules; and the deviation distribution shows two extremes: deviations of 20 molecules exceed 7 kcal·mol^−1^, whereas the deviations of the other 27 molecules are less than 3 kcal·mol^−1^ (There are 53 N-NO bond molecules in total). In addition, for some calculations, the homolysis BDE are dramatically underestimated (the absolute deviations of the DFT calculated homolysis BDE exceed 10 kcal·mol^−1^). The deviations of the calculated and experimental homolysis BDE for the O-NO bond carrier molecule homolysis BDE are relatively large, and the MAD is 5.01 kcal·mol^−1^. The deviations for the C-NO bond homolysis BDE of the carrier molecules are the largest and all of the homolysis BDE are underestimated (MAD is 7.41 kcal·mol^−1^). This is consistent with the results of the Guo group [45], who found that the DFT calculations in a vacuum tend to underestimate the homolysis BDE of Y-NO bond carrier molecules. The results from B3LYP/STO-3G are obviously worse than those from B3LYP/6-31G(d), especially for the S-NO and O-NO bond molecular carriers. The results for the S-NO homolysis BDE have the largest MAD (20.67 kcal·mol^−1^), which is exactly opposite to the results from 6-31G(d), which has the smallest MAD among the four types of Y-NO bonds. This indicates that the polarization function may be obligatory for the S-NO BDE calculations.

### 4.2. SOFMNN Calculation Results

Descriptor selection is a significant step for neural networks, but reports on this topic are scarce [14,15]. In this study, twelve molecular descriptors for each molecule are used. Twelve may seem a small number, but if we exhaust all combinations of these descriptors, there are *C*_12_^1^+*C*_12_^2^ ···+*C*_12_^12^=4095 options. Therefore, if there are hundreds of descriptors (*n*), it is impossible to consider all of the combinations (2*^n^* − 1) without the appropriate methods. The SOFMNN clustering analysis is able to classify similar molecular descriptors into a group; one or several typical molecular descriptors will be selected to represent the group according to the correlation analysis for descriptors and experimental values, considerably reducing the number of descriptors. Through SOFMNN clustering analysis and correlation analysis, subjective selection and bias on molecular descriptors can be avoided and molecular descriptors with the same properties will not be chosen repeatedly. Signals extracted from molecular descriptors can stand out from the noise; therefore, the neural network is more efficient and accurate than the neural network with full molecular descriptors.

**Figure 3:**
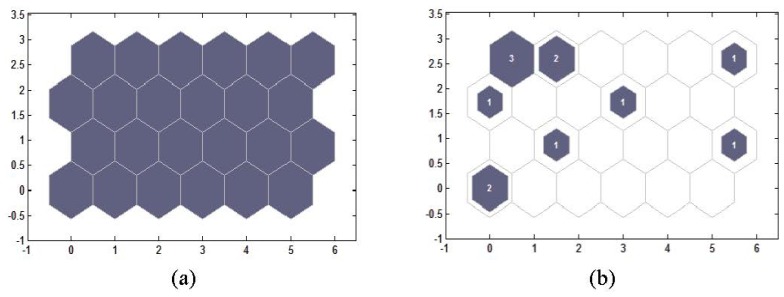
(**a**) The topology structure of the competitive layer; (**b**) Distances of neighbor neurons.

SOFMNN clustering analysis for the molecular descriptors is illustrated by the B3LYP/6-31G(d) calculation results. When twelve molecular descriptors (ΔH_homo_, Q_Y_, Q_N_, Q_O_, N_X_, μ, α, E_HOMO-1_, E_HOMO_, E_LUMO_, E_LUMO+1_ and ΔE) are taken as the input of SOFMNN, the input layer of SOFMNN contains twelve neurons, and a 6 × 4 pattern is adopted in the network structure of the competitive layer (Figure 3a). The number of neurons grows gradually from the bottom left to the top right, *i.e.*, the number of the neurons at the bottom left is 1, and the number on the top right is 24. In Figure 3b, the blue neurons are those that won in competition, and the numbers refer to how many times the neuron has won. The clustering analysis results are reported in Table 2. When the training step is set to 10, ΔH_homo_, N_X_ and α belong to one group, μ itself becomes one group, and all other molecular descriptors are clustered into one group. Similarly, when the training step is set to 30, 50, and 100, the preliminary clustering is performed for the descriptors, but the cluster is not accurate enough because the training steps are not sufficient and the results are not stable. When the number of training steps increases to 1,000, the calculated results of SOFMNN only show small differences when compared to 200 or 500 training steps, *i.e.*, when the training step reaches 500, the clustering results by SOFMNN become steady, and the corresponding clustering number of the twelve molecular descriptors computed by SOFMNN are 16, 13, 1, 19, 12, 8, 24, 19, 19, 20, 20 and 1, respectively. This suggests that the SOFMNN classifies twelve descriptors into eight groups in total: ΔH_homo_, Q_Y_, N_X_, μ and α as five independent groups, Q_N_ and ΔE as one group, Q_O_, E_HOMO-1_ and E_HOMO_ as one group, and E_LUMO_ and E_LUMO+1_ as another group. For groups with more than one descriptor, selection is made according to the correlation analysis results, so Q_N_, E_HOMO_ and E_LUMO_, are chosen because of their higher correlation coefficient. These three descriptors, together with the five independent molecular descriptors (ΔH_homo_, Q_Y_, N_X_, μ and α) are chosen to represent the major characteristics of the Y-NO bond homolysis BDE and are taken as the final inputs of RBFNN. With the same procedure, the nine descriptors ΔH_homo_, Q_Y_, Q_N_, E_HOMO_, N_X_, μ, α, E_LUMO_ and ΔE obtained by B3LYP/STO-3G are selected for the final inputs of RBFNN.

In the SOFMNN calculation, only one neuron wins each time. Its weight and the corresponding weights of its peripheral neurons are adjusted synchronously, and the weights of the neurons change in favor of winning the competition. At the same time, SOFMNN reduces the neighborhood area gradually and starts to repulse its neighbor neurons. The mode combining cooperation with competition allows SOFMNN to acquire superior performance and significantly improves the learning ability and generalization of the neural network. After running the SOFMNN program, the resulting labels are likely different because the excited neurons are different each time, but the final clustering result does not change no matter which neuron is excited.

### 4.3. RBFNN Calculation Results

As mentioned above, eight descriptors (ΔH_homo_, Q_Y_, N_X_, μ, α, Q_N_, E_HOMO_ and E_LUMO_) for B3LYP/6-31G(d) and nine descriptors (ΔH_homo_, Q_Y_, Q_N_, E_HOMO_, N_X_, μ, α, E_LUMO_ and ΔE) for B3LYP/STO-3G selected by SOFMNN clustering analysis and correlation analysis were taken as the RBFNN final inputs. These inputs of RBFNN must be normalized to make the learning and training process easier because the magnitude of the raw data may vary widely if very different raw data are input directly into the neural network. Data with large fluctuations might monopolize the RBFNN learning process, and the network may fail to reflect small changes in data.

In RBFNN, the value of spread is increased from 0.2 to 3 by the constant with a variation of 0.2. The optimal neural network output can be decided during the variation of spread. For DFT-RBFNN and DFT-SOFM-RBFNN methods, the best results of regression estimation are achieved when the values of spread are 0.6 and 0.8, respectively.

**Figure 4:**
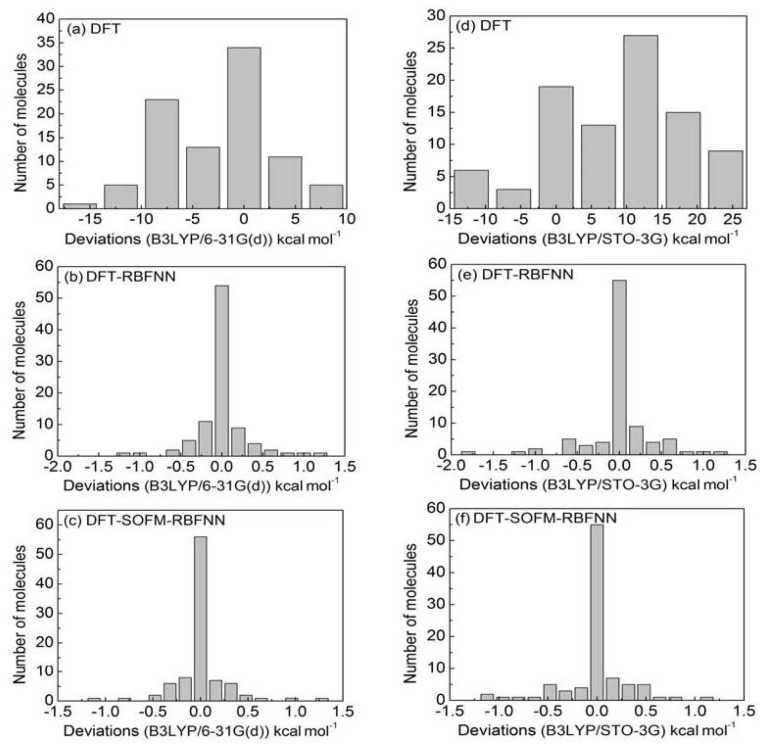
The histograms of deviations between the different calculated homolysis BDE and the experimental values for 92 organic molecules, (**a**) B3LYP/6-31G(d); (**b**) B3LYP/6-31G(d)-RBFNN; (**c**) B3LYP/6-31G(d)-SOFM-RBFNN methods; (**d**–**f**) are the deviations when changing the corresponding basis set from 6-31G(d) to STO-3G.

Figure 4 shows the histograms of deviations between the computed homolysis BDE values and the experimental BDE values. The Figure 4(a–c) presents the histograms of deviations for B3LYP/6-31G(d), B3LYP/6-31G(d)-RBFNN and B3LYP/6-31G(d)-SOFM-RBFNN, respectively, and Figure 4(d–f) are the corresponding deviations of the B3LYP/STO-3G calculations. The deviation in Figure 4a distributions for the homolysis BDE of 92 organic molecules from B3LYP/6-31G(d) calculations are broad and have large systematic deviations, but most are distributed near 0 (34 molecules), which suggests that the accuracy of the results obtained by B3LYP/6-31G(d) is not poor (MAD 4.45 kcal−mol^−1^). However, the deviations for the homolysis BDE by B3LYP/STO-3G in Figure 4d are much worse (the MAD is 10.53 kcal−mol^−1^) than those of the B3LYP/6-31G(d) calculation, and most of the deviations are approximately 12 kcal−mol^−1^ (27 molecules). The results shown in Figure 4(b,e), denoted by DFT-RBFNN, are the DFT results corrected by RBFNN with twelve molecular descriptors (without selection) as inputs. The deviations of the DFT-RBFNN results are remarkably reduced after RBFNN correction. The MADs for the basis sets 6-31G(d) and STO-3G decrease to 0.17 and 0.21 kcal−mol^−1^, respectively; the deviation ranges are narrowed to −1.2–1.3 kcal·mol^−1^ and −1.8–1.2 kcal·mol^−1^; and the deviation distributions are Gaussian curves, indicating that systematic errors have been removed. The DFT-RBFNN effectively improves the accuracy of the DFT calculations.

If we use fewer molecular descriptors, how many descriptors should we choose and which ones should be chosen? These questions can be answered by SOFMNN coupled with correlation analysis. Figure 4(c,f) shows the histograms of deviations for B3LYP with two basis sets corrected by RBFNN with the SOFMNN classified descriptors as inputs, and the method is denoted DFT-SOFM-RBFNN. Calculations are performed to improve DFT calculations, employing these selected descriptors as inputs of RBFNN. In Figure 4(c,f), the deviations of DFT-SOFM-RBFNN are further improved compared with DFT-RBFNN, although the difference is slight. The ranges of deviations are −1.2–1.2 kcal·mol^−1^ and −1.2–1.1 kcal·mol^−1^ and the MADs are 0.15 and 0.18 kcal·mol^−1^ for the 6-31G(d) and STO-3G basis sets, respectively. When regarding only improvements to the accuracy, the significance of SOFMNN is unclear because DFT-RBFNN is already sufficiently accurate, but SOFMNN increases the calculation efficiency and solves mass descriptor problems, very well when many descriptors are used. Although the improvement of accuracy compared with DFT-RBFNN is slight, chemical accuracy (1 kcal·mol^−1^) is achieved for all 92 Y-NO homolysis BDE calculation results, which is a very important result. Surprisingly, the homolysis BDE by B3LYP/STO-3G after correction are comparable to those by B3LYP/6-31G(d), even with the raw MAD (10.53 kcal·mol^−1^) of STO-3G being much worse than that (4.45 kcal·mol^−1^) of 6-31G(d). With the minimal basis set STO-3G, we can save much time and many resources while retaining the ability to perform calculations for large molecules.

During this study, we considered the extrapolation of the method to larger molecules and molecules with more types of elements as well as to different Y-NO bonds in addition to the four types in this dataset, so we preferred descriptors that were independent of the elemental types. After establishing the DFT-SOFM-RBFNN method, some molecules were used to test the ability to extrapolate. The structures of the molecules and the calculation results are shown in Table 3. Six extrapolation test molecules contained Si-NO bonds and fluorine, which were not included in original dataset. The DFT-SOFM-RBFNN results show that deviations of the DFT calculations for test molecules are reduced dramatically and reach the same accuracy as the 92 organic Y-NO bond molecules, particularly for B3LYP/STO-3G calculation results with large calculation deviations, which gives us more confidence in the predictive ability of this method.

The excellent performance of the DFT-SOFM-RBFNN method benefits from the combined advantages of all the methods. DFT molecular descriptors represent the physical essence of the homolysis BDE; the RBFNN is independent of the initial weights and thresholds, converges quickly to global minima, has few parameters that must be adjusted, shows great capacity for reverse redundancy and fault tolerance and possesses a built-in nonlinear model capable of carrying out calculations with a partial response. As a result of the SOFMNN cluster analysis, the significant features of the descriptors have been discovered and the number of descriptors can be narrowed down, so that the accuracy and efficiency of RBFNN calculations are improved. The combined DFT-SOFM-RBFNN method improves the DFT calculations and develops new applications in chemistry for SOFMNN and RBFNN.

To compare the DFT-SOFM-RBFNN calculations with more sophisticated DFT calculations with a larger basis set, the M06-2X/6-311 + G(2d,p) calculations with or without the solvent effect are performed for the four smallest molecules from each type of Y-NO molecule. The results are listed in Table 4. As shown in Table 4, the BDE calculations are improved by the M06-2X/6-311 + G(2d,p) calculation compared to the B3LYP/6-31G(d) calculations, but high accuracy cannot be reached. The solvent effect by the polarizable continuum model (PCM) on the BDE is adopted. The results show that the solvent effects are small (<2 kcal·mol^−1^) and uncertain for improvement of BDE calculations, and the chemical accuracy cannot be reached even when considering the solvent effects. This further exhibits the high efficiency and accuracy of the proposed DFT-SOFM-RBFNN method.

## 5. Conclusions

Recently, artificial intelligence, or “machine learning,” has begun to be employed to solve first-principles/quantum chemical calculation problems in a simple and efficient manner; therefore, they can reach statistically interesting problems rather than simply solving wave functions. In this study, the accuracy of the DFT calculations for the homolysis BDE of 92 organic NO carrier molecules was improved by the proposed DFT-SOFM-RBFNN method, which combines first-principles DFT and artificial intelligence SOFMNN and RBFNN. The DFT computes the molecular descriptors/quantum mechanical descriptors, the SOFMNN performs cluster analysis to classify molecular descriptors, and the correlation analysis selects descriptor from each classified group; thus, subjective opinions on and the biases of molecular descriptors can be avoided. Thereafter, RBFNN uses these selected molecular descriptors as inputs to correct the DFT-calculated homolysis BDE. The DFT calculations are performed by B3LYP with two basis sets, the minimal basis set STO-3G and the medium size basis set 6-31G(d). In total, twelve descriptors are obtained, eight and nine groups are categorized by SOFMNN for descriptors acquired with the B3LYP/6-31G(d) and B3LYP/STO-3G basis sets, respectively. After the final RBFNN calculations, chemical accuracy (≤1 kcal·mol^−1^) is achieved for all DFT-calculated homolysis BDE of 92 NO carrier molecules. The overall MADs of the homolysis BDE calculated by the B3LYP method with the 6-31G(d) and STO-3G basis sets decrease from 4.45 to 0.15 kcal·mol^−1^ and from 10.53 to 0.18 kcal·mol^−1^, respectively. Although the raw MAD by B3LYP/STO-3G was much worse than that of B3LYP/6-31G(d), high accuracy for B3LYP/STO-3G has yet to be obtained. The minimal basis set DFT-SOFM-RBFNN could apply to fairly large molecules; additionally, the molecular descriptors used are general, which makes the method easy to use and further extrapolate to various system; extrapolation tests proved that high-accuracy results can be achieved for molecules with different types of Y-NO bond and systems including atoms not already in the database. In particular, the high-accuracy result obtained in the study is practically important for the design of new types of NO-releasing drug molecules. We firmly believe that DFT-SOFM-RBFNN can calculate not only the homolysis BDE but also other interesting properties such as bond heterolysis energy, optical properties, power conversion efficiency, and further research is ongoing.

## Supplementary Materials



## Figures and Tables

**Table 1:** Deviations between experimental and calculated values of 92 organic molecules from different methods, reported in kcal·mol^−1^.

No.	B3LYP/6-31G(d)	B3LYP/STO-3G	DFT-RBFNN		DFT-SOFM-RBFNN	
	
			6-31G(d)	STO-3G	6-31G(d)	STO-3G
1	−17.17	−6.89	−0.12	−1.84	−0.04	−1.18
2	−7.88	2.66	0.46	0.12	0.38	0.22
3	−9.31	0.85	−0.48	−0.65	−0.38	−0.58
4	−9.29	1.27	−0.03	−0.02	−0.01	−0.01
5	−9.77	0.14	0.00	−0.01	0.00	0.00
6^a^	−9.13	1.04	−0.40	−0.53	−0.34	−0.46
7	−9.01	1.11	0.05	−0.01	0.03	0.01
8	−12.53	0.28	−0.03	0.00	−0.01	0.00
9	−13.13	−3.06	0.00	0.00	0.00	0.00
10	−10.9	−0.51	−0.01	−0.01	0.00	0.00
11	2.16	12.31	0.07	0.02	0.04	0.01
12	2.70	13.23	0.58	0.81	0.55	0.68
13	1.72	12.17	−0.34	0.42	−0.34	0.23
14	−0.39	10.26	−0.10	0.03	−0.06	0.01
15	−1.56	10.1	0.00	0.01	0.00	0.01
16	1.69	11.63	0.00	0.01	0.00	0.00
17	2.00	12.39	−0.20	0.25	−0.23	0.13
18	−8.37	2.73	−0.16	0.05	−0.06	0.03
19	−7.30	4.12	−0.28	−0.02	−0.21	−0.01
20^a^	−6.93	4.16	−0.22	−0.47	−0.21	−0.41
21	−7.68	3.96	0.29	0.01	0.27	0.00
22	−10.58	0.56	0.00	0.00	0.00	0.00
23	−2.11	8.33	0.01	−0.93	0.06	−0.75
24	3.45	12.33	0.35	0.67	0.19	0.45
25	−8.07	3.05	−0.53	−0.21	−0.51	−0.18
26	−7.90	3.23	0.28	0.18	0.29	0.17
27^a^	−8.60	2.58	−0.42	−0.01	−0.38	−0.01
28	−8.22	4.07	0.01	0.00	0.00	0.00
29	−4.97	6.77	0.00	0.00	0.00	0.00
30	1.87	−11.2	0.00	0.02	0.00	0.01
31^a^	1.97	−11.27	−0.05	0.00	−0.04	0.00
32	0.33	−12.53	−0.01	−0.03	0.00	−0.02
33^a^	1.91	−6.79	0.04	−0.03	0.03	−0.03
34	0.74	−11.6	0.00	0.00	0.00	0.00
35	1.92	−10.83	0.18	0.01	0.15	0.01
36	0.62	−14	−0.18	0.00	−0.15	0.00
37	1.16	10.52	0.00	0.00	0.00	0.00
38	0.76	11.2	0.14	0.12	0.10	0.10
39	0.29	11.06	−0.05	−0.09	−0.07	−0.08
40	−0.36	10.68	−0.06	−0.39	−0.05	−0.36
41	−0.41	11.52	0.00	0.00	0.00	0.00
42	−0.04	11.72	0.02	0.40	0.01	0.37
43	−0.26	10.28	0.04	−0.05	0.04	−0.03
44^a^	−1.14	11.08	1.01	0.95	0.92	0.84
45	−0.97	9.89	0.00	0.00	0.00	0.00
46	0.03	12.03	0.00	0.00	0.00	0.00
47	0.87	10.84	0.02	0.04	0.01	0.02
48	−1.67	8.65	0.00	0.00	0.00	0.00
49	−3.41	8.59	−0.01	−0.03	0.00	−0.02
50	7.47	−0.71	−0.01	0.01	0.01	0.01
51	5.60	−0.55	0.00	0.00	0.00	0.00
52	7.03	−1.38	0.03	0.00	0.01	0.00
53	6.33	−2.14	−0.01	−0.01	−0.01	−0.01
54	−2.62	15.71	0.00	0.00	0.00	0.00
55	−2.88	15.23	0.12	0.28	0.08	0.25
56	−3.88	14.1	−0.12	−0.28	−0.08	−0.25
57	−3.89	13.76	0.00	−0.01	0.00	−0.01
58^a^	−7.57	9.35	0.00	0.00	0.00	0.00
59	−4.88	12.76	1.26	1.19	1.20	1.14
60	−7.33	9.84	−1.20	−1.15	−1.16	−1.12
61	−6.90	10.9	0.17	0.26	0.20	0.28
62	6.39	18.5	0.00	0.00	0.00	0.00
63	4.12	17.94	0.00	0.38	0.00	0.35
64	−9.96	16.41	0.00	−0.37	0.00	−0.34
65	4.19	15.06	0.00	−0.01	0.00	−0.01
66	0.55	14.42	0.00	0.00	0.00	0.00
67	−3.51	19.3	−0.60	−0.52	−0.47	−0.43
68	−2.46	21.15	−0.93	−0.93	−0.85	−0.90
69	0.27	22.96	0.51	0.57	0.44	0.54
70	0.05	22.7	0.07	0.50	0.04	0.47
71^a^	2.43	22.6	0.19	0.18	0.16	0.14
72	0.20	19.63	0.01	0.00	0.00	0.00
73	−0.88	20.53	−0.16	−0.52	−0.09	−0.48
74	7.91	19.5	0.02	0.03	0.01	0.02
75	−0.36	22.56	0.38	0.39	0.39	0.40
76	2.96	21.38	0.00	0.00	0.00	0.00
77	1.69	22.06	0.83	0.53	0.61	0.43
78	2.77	21.23	0.00	0.01	0.00	0.01
79	2.52	20.27	0.21	0.00	0.13	0.00
80	0.84	19.65	0.01	−0.01	0.00	−0.01
81	1.17	21.22	0.00	0.00	0.00	0.00
82	0.68	20.49	−0.21	0.00	−0.13	0.00
83^a^	−2.03	16.73	−0.27	−0.57	−0.26	−0.56
84^a^	−0.24	18.15	0.27	0.57	0.26	0.56
85^a^	−7.63	2.33	−0.04	0.02	−0.03	0.02
86	−4.58	6.59	0.00	0.00	0.00	0.00
87	−7.16	5.16	0.48	0.16	0.36	0.12
88	−8.00	2.5	0.02	0.10	0.01	0.07
89	−3.70	11.26	0.00	0.00	0.00	0.00
90	−10.85	0.62	−0.49	−0.26	−0.37	−0.18
91^a^	−8.77	5.98	−0.16	−0.17	−0.13	−0.13
92	−8.61	1.34	0.00	0.00	0.00	0.00

a The molecules belong to the test set.

**Table 2:** SOFMNN clustering analysis results for twelve molecular descriptors.

DFT	Training Steps	Clustering Analysis											

		ΔH_homo_	Q_Y_	Q_N_	Q_O_,	N_X_	μ	α	E_HOMO-1_	E_HOMO_	E_LUMO_	E_LUMO+1_	ΔE
B3LYP/6-31G(d)	10	24	1	1	1	24	4	24	1	1	1	1	1
	30	5	13	13	13	24	19	24	13	13	13	13	13
	50	4	12	6	12	1	21	1	12	12	12	12	12
	100	19	12	10	12	3	22	1	12	12	11	11	10
	200	16	1	8	1	11	19	24	1	1	2	2	8
	500	16	13	1	19	12	8	24	19	19	20	20	1
	1000	16	13	20	13	23	2	24	13	13	14	14	20

B3LYP/STO-3G	10	2	1	1	1	24	1	24	1	1	1	1	1
	30	23	1	7	1	24	5	24	1	1	1	2	7
	50	21	1	1	1	6	13	12	1	1	1	1	1
	100	21	7	19	7	24	3	12	7	7	14	19	19
	200	5	7	19	1	24	3	22	1	1	13	14	15
	500	4	16	19	21	24	8	12	21	21	20	19	13
	1000	10	13	15	19	24	2	12	19	19	20	15	21

**Table 3:** The extrapolation test for the DFT-SOFM-RBFNN method. (kcal·mol^−1^).

No.	Structures	Expt.	B3LYP/6-31G(d)	DFT-SOFM-RBFNN 6-31G(d)	B3LYP/STO-3G	DFT-SOFM-RBFNN STO-3G
1	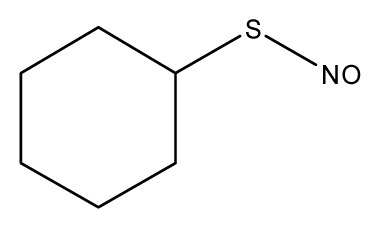	31.6	29.02	30.49	48.10	30.56
2	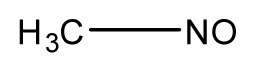	41.1	38.55	40.95	49.4	40.59
3	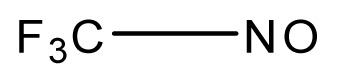	39.9	37.67	39.90	32.47	39.90
4	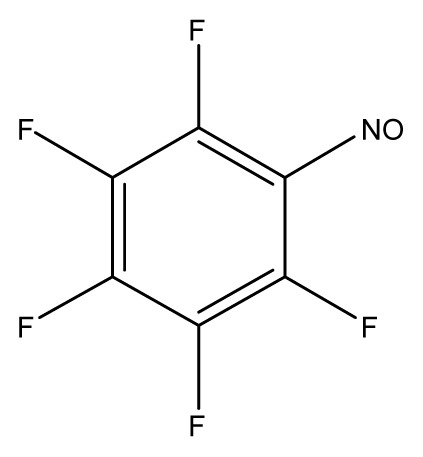	50.5	50.34	50.48	60.6	51.12
5	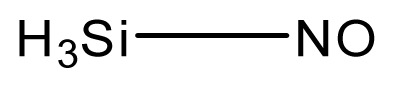	37.8	27.04	37.85	48.8	37.98
6	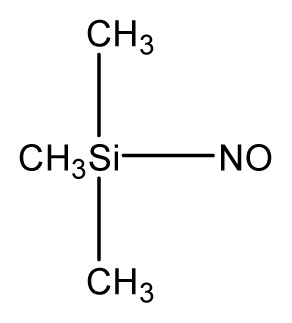	44.8	34.65	44.76	50.58	44.62

**Table 4:** The deviations of calculation methods (kcal·mol^−1^).

NO.	DFT-SOFM-RBFNN^a^	M06-2X/6-311 + G(2d,p)	M06-2X/6-311 + G(2d,p) (PCM)	B3LYP/6-31G(d)
39	−0.1	3.6	2.4	0.29
59	1.2	1.5	0.8	−4.9
76	0.0	4.2	4.2	3.0
91	−0.1	−2.2	−4.1	−8.7

a DFT-SOFM-RBFNN is based on B3LYP/6-31G(d) calculations.
