# Supplementary material for: A Promising Tool to Achieve Chemical Accuracy for Density Functional Theory Calculations on Y-NO Homolysis Bond Dissociation Energies
Source: Int J Mol Sci. 2012 Jun 28;13(7):8051–70. doi: 10.3390/ijms13078051 (PMC3430220; doi:10.3390/ijms13078051)
Supplement: Supplementary file 1 [file ijms-13-08051-s001.pdf]

A Promising Tool to Achieve Chemical Accuracy for Density Functional Theory Calculations on Y-NO Homolysis Bond Dissociation Energies

Supplementary Materials

Table 1. Calculated and experimental homolysis BDE of Y-NO (Y = C, N, O, S) bonds and molecular descriptors [a].

No. [b]	Expt. [c]	ΔH_{homo} [d]	Q_Y	Q_N	Q_O	N_X	μ	α	E_{Homo-1}	E_{Homo}	E_{Lumo}	E_{Lumo+1}	ΔE
1	43.8	26.63	−0.309	0.222	−0.325	87	1.83	117.55	−0.2466	−0.2275	−0.0744	−0.0114	0.1532
2	36.1	28.22	−0.311	0.223	−0.324	79	0.91	111.89	−0.2493	−0.2457	−0.0761	−0.0190	0.1696
3	38.3	28.99	−0.312	0.224	−0.323	71	0.46	98.39	−0.2580	−0.2494	−0.0781	−0.0223	0.1713
4	37.6	28.31	−0.314	0.231	−0.324	87	1.53	111.82	−0.2584	−0.2542	−0.0855	−0.0344	0.1687
5	39.2	29.43	−0.318	0.236	−0.318	93	4.54	117.63	−0.2838	−0.2686	−0.1039	−0.0916	0.1648
6	34.5	25.37	−0.613	0.230	−0.341	145	2.81	182.34	−0.2432	−0.2360	−0.0556	−0.0086	0.1804
7	35.0	25.99	−0.614	0.231	−0.340	137	2.89	168.56	−0.2501	−0.2397	−0.0578	−0.0115	0.1819
8	36.2	23.67	−0.627	0.251	−0.336	153	3.65	182.09	−0.2476	−0.2432	−0.0666	−0.0196	0.1766
9	40.4	27.27	−0.615	0.239	−0.332	159	6.30	189.00	−0.2736	−0.2582	−0.0968	−0.0707	0.1614
10	36.2	25.30	−0.614	0.234	−0.338	171	3.60	190.40	−0.2498	−0.2425	−0.0652	−0.0231	0.1773
11	21.4	23.56	−0.247	0.213	−0.368	105	4.59	157.91	−0.2261	−0.2146	−0.0500	−0.0147	0.1646
12	21.4	24.10	−0.248	0.214	−0.366	97	3.78	152.40	−0.2278	−0.2213	−0.0523	−0.0184	0.1690
13	22.6	24.32	−0.250	0.216	−0.365	89	3.55	138.43	−0.2299	−0.2248	−0.0545	−0.0211	0.1702
14	24.1	23.71	−0.252	0.220	−0.361	105	2.73	150.61	−0.2373	−0.2318	−0.0628	−0.0309	0.1690
15	24.3	22.74	−0.256	0.226	−0.353	111	5.18	161.02	−0.2497	−0.2437	−0.0997	−0.0674	0.1440
16	21.0	22.69	−0.245	0.209	−0.376	121	5.80	178.22	−0.2209	−0.2028	−0.0445	−0.0135	0.1584
17	22.3	24.30	−0.249	0.215	−0.367	97	3.44	151.74	−0.2281	−0.2221	−0.0530	−0.0193	0.1691
18	28.3	19.93	−0.326	0.218	−0.338	103	3.29	136.91	−0.2379	−0.2228	−0.0567	−0.0045	0.1661
19	28.7	21.40	−0.327	0.219	−0.337	95	2.37	131.36	−0.2430	−0.2373	−0.0583	−0.0077	0.1791
20	29.1	22.17	−0.328	0.220	−0.336	87	2.54	117.73	−0.2507	−0.2402	−0.0604	−0.0108	0.1798
21	29.2	21.52	−0.330	0.226	−0.337	103	3.85	131.41	−0.2511	−0.2463	−0.0683	−0.0225	0.1781
22	33.1	22.52	−0.333	0.232	−0.331	109	6.56	137.77	−0.2664	−0.2592	−0.0970	−0.0752	0.1622
23	27.5	25.39	−0.322	0.219	−0.345	95	2.41	128.37	−0.2449	−0.2395	−0.0585	−0.0139	0.1810
24	23.1	26.55	−0.332	0.234	−0.334	103	1.55	127.84	−0.2527	−0.2444	−0.0631	−0.0253	0.1813
25	30.3	22.23	−0.330	0.228	−0.336	121	4.08	137.71	−0.2481	−0.2459	−0.0683	−0.0235	0.1776
26	29.4	21.50	−0.330	0.226	−0.337	121	3.75	139.32	−0.2492	−0.2441	−0.0682	−0.0230	0.1759
27	30.5	21.90	−0.330	0.227	−0.335	109	3.72	147.05	−0.2515	−0.2480	−0.0736	−0.0553	0.1744
28	26.6	18.38	−0.327	0.226	−0.348	127	3.31	174.50	−0.2394	−0.2322	−0.0553	−0.0030	0.1769
29	25.4	20.43	−0.322	0.218	−0.338	175	2.07	247.88	−0.2389	−0.2371	−0.0619	−0.0130	0.1752
30	33.7	35.57	−0.489	0.218	−0.367	105	5.11	128.42	−0.2500	−0.2435	−0.0595	−0.0395	0.1840
31	33.4	35.37	−0.492	0.221	−0.363	97	5.10	122.36	−0.2690	−0.2467	−0.0633	−0.0466	0.1833
32	34.9	35.23	−0.493	0.221	−0.361	89	4.54	108.58	−0.2783	−0.2491	−0.0662	−0.0499	0.1829
33	33.0	34.91	−0.495	0.220	−0.356	105	3.11	122.51	−0.2757	−0.2545	−0.0730	−0.0591	0.1815
34	33.9	34.64	−0.497	0.220	−0.349	111	2.00	125.62	−0.2986	−0.2629	−0.1125	−0.0797	0.1505
35	33.0	34.92	−0.495	0.221	−0.356	123	3.23	130.46	−0.2702	−0.2542	−0.0729	−0.0591	0.1813
36	33.7	34.32	−0.501	0.228	−0.349	121	3.73	132.97	−0.2716	−0.2568	−0.0733	−0.0694	0.1835
37	28.7	29.86	−0.219	0.233	−0.355	77	4.28	117.41	−0.2290	−0.2198	−0.0760	−0.0191	0.1438
38	28.6	29.36	−0.221	0.235	−0.351	69	3.13	111.26	−0.2372	−0.2276	−0.0779	−0.0198	0.1497
39	29.0	29.29	−0.223	0.237	−0.348	61	2.85	97.11	−0.2465	−0.2297	−0.0802	−0.0233	0.1495
40	29.8	29.44	−0.224	0.241	−0.343	77	2.20	111.05	−0.2467	−0.2414	−0.0893	−0.0360	0.1521
41	29.3	28.89	−0.229	0.249	−0.329	83	4.00	116.51	−0.2715	−0.2541	−0.1089	−0.0828	0.1451
42	28.5	28.43	−0.225	0.243	−0.341	77	3.79	110.11	−0.2569	−0.2338	−0.0891	−0.0356	0.1447
43	29.7	29.40	−0.224	0.241	−0.343	95	2.19	119.13	−0.2433	−0.2410	−0.0893	−0.0360	0.1518
44	22.9	21.76	−0.265	0.217	−0.369	103	3.79	155.37	−0.2333	-0.2254	−0.0486	−0.0139	0.1767
45	13.6	12.63	−0.230	0.208	−0.376	95	3.04	143.30	−0.2346	−0.2119	−0.0623	−0.0231	0.1496

Table 1. *Cont.*

No. [b]	Expt. [c]	ΔH_{homo} [d]	Q_Y	Q_N	Q_O	N_X	μ	α	E_{Homo-1}	E_{Homo}	E_{Lumo}	E_{Lumo+1}	ΔE
46	19.2	19.23	−0.271	0.222	−0.369	101	3.70	165.31	−0.2299	−0.2158	−0.0573	−0.0476	0.1585
47	27.4	28.27	−0.210	0.229	−0.362	87	3.19	142.90	−0.2329	−0.2307	−0.0751	−0.0406	0.1557
48	28.3	26.63	−0.212	0.233	−0.353	155	0.72	189.38	−0.2452	−0.2386	−0.0898	−0.0596	0.1488
49	29.7	26.29	−0.209	0.223	−0.362	99	3.44	152.06	−0.2377	−0.2152	−0.0721	−0.0161	0.1431
50	13.2	20.67	−0.516	0.240	−0.332	121	5.38	156.76	−0.2605	−0.2475	−0.0697	−0.0523	0.1778
51	12.4	18.00	−0.513	0.237	−0.335	137	6.62	176.96	−0.2454	−0.2315	−0.0661	−0.0483	0.1654
52	13.1	20.13	−0.517	0.242	−0.331	137	4.85	171.04	−0.2594	−0.2532	−0.0759	−0.0586	0.1772
53	14.5	20.83	−0.518	0.241	−0.329	143	3.23	186.80	−0.2584	−0.2540	−0.0809	−0.0706	0.1732
54	32.5	29.88	−0.482	0.419	−0.205	79	4.05	111.15	−0.2672	−0.2370	−0.1077	−0.0420	0.1293
55	32.8	29.92	−0.483	0.421	−0.200	71	3.46	103.71	−0.2637	−0.2570	−0.1116	−0.0490	0.1454
56	33.9	30.02	−0.485	0.424	−0.195	63	2.84	89.12	−0.2682	−0.2658	−0.1150	−0.0522	0.1508
57	34.3	30.41	−0.489	0.427	−0.188	97	1.43	111.98	−0.2795	−0.2591	−0.1215	−0.0619	0.1375
58	38.6	31.03	−0.496	0.436	−0.171	85	2.96	107.57	−0.2964	−0.2920	−0.1352	−0.1074	0.1568
59	35.0	30.12	−0.491	0.421	−0.193	31	2.09	39.15	−0.3068	−0.2791	−0.1159	0.0010	0.1632
60	37.9	30.57	−0.488	0.420	−0.195	39	2.04	50.08	−0.3058	−0.2772	−0.1146	0.0031	0.1626
61	36.7	29.80	−0.488	0.420	−0.196	47	2.14	60.61	−0.3048	−0.2737	−0.1142	0.0011	0.1595
62	33.7	40.09	−0.380	0.383	−0.325	73	3.67	102.43	−0.2589	−0.2246	−0.0688	−0.0089	0.1559
63	33.7	37.82	−0.379	0.384	−0.323	65	3.06	96.97	−0.2564	−0.2425	−0.0706	−0.0160	0.1719
64	35.0	25.04	−0.380	0.385	−0.322	57	2.68	83.49	−0.2591	−0.2515	−0.0725	−0.0192	0.1790
65	36.2	40.39	−0.382	0.388	−0.318	91	1.54	104.40	−0.2738	−0.2482	−0.0785	−0.0326	0.1698
66	36.2	36.75	−0.388	0.394	−0.308	79	3.17	102.33	−0.2882	−0.2792	−0.0977	−0.0898	0.1815
67	21.0	17.49	0.289	0.054	−0.229	73	3.68	111.93	−0.2520	−0.2181	−0.0905	−0.0398	0.1276
68	21.4	18.94	0.297	0.054	−0.225	65	2.91	105.93	−0.2532	−0.2279	−0.0942	−0.0439	0.1337
69	19.4	19.67	0.300	0.055	−0.222	57	2.36	91.82	−0.2556	−0.2333	−0.0973	−0.0465	0.1360
70	19.2	19.25	0.296	0.062	−0.214	73	0.52	106.01	−0.2618	−0.2381	−0.1047	−0.0568	0.1333
71	18.6	21.03	0.303	0.075	−0.197	79	3.05	113.42	−0.2737	−0.2576	−0.1206	−0.1006	0.1370
72	23.4	23.60	0.318	0.012	−0.245	145	2.85	221.05	−0.2407	−0.2280	−0.0898	−0.0303	0.1382
73	20.9	20.02	0.298	0.065	−0.211	73	2.08	104.13	−0.2624	−0.2415	−0.1058	−0.0575	0.1357
74	19.3	27.21	0.292	0.077	−0.212	73	3.13	102.84	−0.2606	−0.2369	−0.1006	−0.0593	0.1363
75	19.9	19.54	0.302	0.053	−0.224	65	2.62	104.38	−0.2536	−0.2303	−0.0953	−0.0443	0.1351
76	25.0	27.96	0.316	−0.005	−0.247	49	2.76	73.43	−0.2584	−0.2331	−0.0887	−0.0126	0.1445
77	17.2	18.89	0.310	0.057	−0.231	73	3.50	108.44	−0.2391	−0.2188	−0.0870	−0.0415	0.1318
78	24.4	27.17	0.310	0.013	−0.243	179	2.14	218.16	−0.2537	−0.2397	−0.0944	−0.0275	0.1452
79	24.3	26.82	0.317	0.009	−0.236	161	4.93	172.55	−0.2702	−0.2519	−0.1066	−0.0373	0.1453
80	26.2	27.04	0.306	0.016	−0.240	171	2.49	197.57	−0.2432	−0.2241	−0.0977	−0.0288	0.1263
81	26.1	27.27	0.313	0.005	−0.242	131	2.22	148.15	−0.2537	−0.2427	−0.0974	−0.0254	0.1453
82	26.6	27.28	0.325	0.003	−0.239	163	4.86	191.09	−0.2583	−0.2448	−0.0994	−0.0299	0.1454
83	29.2	27.17	0.311	0.012	−0.243	163	1.57	190.12	−0.2535	−0.2395	−0.0942	0.0246	0.1452
84	27.4	27.16	0.306	0.017	−0.241	139	1.23	158.73	−0.2539	−0.2394	−0.0939	−0.0252	0.1455
85	28.8	21.17	−0.021	0.126	−0.284	79	2.14	112.96	−0.2611	−0.2274	−0.0916	−0.0653	0.1358
86	29.2	24.62	−0.036	0.139	−0.284	85	2.66	121.41	−0.2621	−0.2295	−0.0946	−0.0621	0.1349
87	27.5	20.34	−0.120	0.173	−0.254	117	3.29	135.55	−0.2727	−0.2310	−0.0915	−0.0139	0.1396
88	27.6	19.60	−0.119	0.151	−0.255	109	0.96	126.38	−0.2794	−0.2307	−0.0931	−0.0111	0.1376
89	26.2	22.5	0.187	0.136	−0.244	69	4.52	82.90	−0.2974	−0.2483	−0.1097	−0.0745	0.1386
90	30.4	19.55	−0.121	0.149	−0.262	101	2.94	115.49	−0.2788	−0.2314	−0.0927	−0.0150	0.1387
91	31.4	22.63	0.177	0.144	−0.239	47	3.99	55.09	−0.3002	−0.2542	−0.1127	−0.0783	0.1415
92	26.3	17.69	−0.118	0.139	−0.261	61	1.77	78.22	−0.2648	−0.2386	−0.1020	−0.0552	0.1366

[a] Unit: ΔH_{homo} (kcal·mol^{-1}), Charge (e), Dipole Moment (debye), Polarizability (a.u.) and Energy (a.u.); [b] 1~53 contain N-NO, 54~66 contain O-NO, 67~84 contain S-NO, 85~92 contain C-NO; [c] Measured in CH3CN at 25 ℃ by titration calorimetry; [d] The calculated homolysis bond dissociation energies are with zero-point energy (ZPE) and thermal corrections to enthalpy at 298K by B3LYP/6-31G(d).

Table 2. Calculated and experimental homolysis BDE of Y-NO (Y = C, N, O, S) bonds and molecular descriptors [a].

No. [b,e]	Expt. [c]	ΔH_{homo} [d]	Q_Y	Q_N	Q_O	N_X	μ	α	E_{Homo-1}	E_{Homo}	E_{Lumo}	E_{Lumo+1}	ΔE
1	43.8	36.91	−0.192	0.092	−0.114	87	1.66	82.95	−0.1477	−0.1352	0.0089	0.0754	0.1441
2	36.1	38.76	−0.193	0.095	−0.111	79	0.68	75.85	−0.1590	−0.1492	0.0047	0.0685	0.1539
3	38.3	39.15	−0.193	0.096	−0.110	71	0.28	67.68	−0.1634	−0.1518	0.0023	0.0657	0.1541
4	37.6	38.87	−0.194	0.102	−0.106	87	2.30	76.28	−0.1716	−0.1623	−0.0087	0.0487	0.1536
5	39.2	39.34	−0.194	0.105	−0.103	93	3.31	84.16	−0.1582	−0.1511	−0.0148	0.0083	0.1363
6	34.5	35.54	−0.304	0.080	−0.127	145	2.14	125.93	−0.1388	−0.1340	0.0221	0.0969	0.1561
7	35.0	36.11	−0.305	0.081	−0.126	137	2.28	117.12	−0.1434	−0.1359	0.0198	0.0940	0.1557
8	36.2	36.48	−0.305	0.086	−0.122	153	4.47	126.71	−0.1519	−0.1457	0.0088	0.0756	0.1545
9	40.4	37.34	−0.304	0.089	−0.118	159	5.54	135.56	−0.1472	−0.1399	−0.0005	0.0282	0.1394
10	36.2	35.69	−0.304	0.083	−0.125	171	3.19	130.31	−0.1401	−0.1392	0.0153	0.0853	0.1545
11	21.4	33.71	−0.164	0.092	−0.124	105	3.20	113.29	−0.1451	−0.1276	0.0204	0.0744	0.1480
12	21.4	34.63	−0.164	0.095	−0.121	97	2.13	106.20	−0.1492	−0.1378	0.0161	0.0684	0.1539
13	22.6	34.77	−0.165	0.096	−0.120	89	1.78	97.73	−0.1517	−0.1408	0.0136	0.0660	0.1544
14	24.1	34.36	−0.165	0.103	−0.115	105	1.36	105.29	−0.1628	−0.1510	0.0026	0.0512	0.1536
15	24.3	34.40	−0.165	0.105	−0.113	111	2.99	115.85	−0.1553	−0.1508	−0.0059	0.0121	0.1449
16	21.0	32.63	−0.163	0.088	−0.129	121	4.29	130.32	−0.1362	−0.1129	0.0268	0.0794	0.1397
17	22.3	34.69	−0.165	0.095	−0.121	97	1.60	105.48	−0.1495	−0.1386	0.0157	0.0680	0.1543
18	28.3	31.03	−0.201	0.094	−0.113	103	2.22	96.81	−0.1462	−0.1255	0.0150	0.0924	0.1405
19	28.7	32.82	−0.202	0.097	−0.110	95	1.73	89.47	−0.1493	−0.1432	0.0112	0.0869	0.1544
20	29.1	33.26	−0.202	0.098	−0.108	87	1.99	81.08	−0.1512	−0.1472	0.0088	0.0847	0.1560
21	29.2	33.16	−0.202	0.103	−0.104	103	4.20	90.09	−0.1603	−0.1568	−0.0026	0.0657	0.1542
22	33.1	33.66	−0.202	0.106	−0.101	109	5.20	98.41	−0.1520	−0.1447	−0.0091	0.0173	0.1356
23	27.5	35.83	−0.211	0.089	−0.118	95	1.60	85.34	−0.1479	−0.1405	0.0201	0.0714	0.1605
24	23.1	35.43	−0.201	0.100	−0.116	103	0.34	86.42	−0.1533	−0.1483	0.0066	0.0583	0.1549
25	30.3	33.35	−0.202	0.101	−0.106	121	2.83	91.31	−0.1552	−0.1527	0.0033	0.0764	0.1560
26	29.4	32.63	−0.202	0.100	−0.107	121	2.89	93.29	−0.1550	−0.1451	0.0042	0.0758	0.1492
27	30.5	33.08	−0.202	0.102	−0.104	109	3.15	102.68	−0.1517	−0.1417	0.0013	0.0406	0.1430
28	26.6	30.67	−0.201	0.092	−0.111	127	2.29	113.58	−0.1446	−0.1394	0.0144	0.0869	0.1538
29	25.4	32.17	−0.197	0.093	−0.118	175	1.58	166.35	−0.1493	−0.1443	0.0115	0.0747	0.1558
30	33.7	22.50	−0.171	0.037	−0.119	105	4.69	104.60	−0.1309	−0.1278	0.0015	0.0500	0.1293
31	33.4	22.13	−0.164	0.031	−0.120	97	3.42	94.68	−0.1348	−0.1319	−0.0036	0.0418	0.1283
32	34.9	22.37	−0.169	0.040	−0.116	89	3.66	88.46	−0.1362	−0.1333	−0.0036	0.0433	0.1297
33	33.0	26.21	−0.168	0.043	−0.112	105	1.65	97.13	−0.1443	−0.1421	−0.0115	0.0314	0.1306
34	33.9	22.30	−0.167	0.044	−0.110	111	2.10	103.10	−0.1468	−0.1445	−0.0140	-0.0065	0.1306
35	33.0	22.17	−0.169	0.041	−0.115	123	2.79	100.75	−0.1391	−0.1364	−0.0067	0.0387	0.1297
36	33.7	19.7	−0.161	0.050	−0.104	121	2.25	103.89	−0.1534	−0.1519	−0.0190	0.0229	0.1330
37	28.7	39.22	−0.134	0.117	−0.101	77	2.20	83.74	−0.1557	−0.1329	−0.0082	0.0714	0.1247
38	28.6	39.80	−0.135	0.120	−0.098	69	1.07	75.94	−0.1676	−0.1542	−0.0119	0.0696	0.1423
39	29.0	40.06	−0.136	0.121	−0.097	61	0.80	67.50	−0.1749	−0.1569	−0.0144	0.0661	0.1426
40	29.8	40.48	−0.135	0.129	−0.090	77	2.22	76.03	−0.1824	−0.1705	−0.0273	0.0482	0.1432
41	29.3	40.82	−0.136	0.132	−0.087	83	3.17	83.22	−0.1540	−0.1468	−0.0326	0.0164	0.1141
42	28.5	40.19	−0.136	0.130	−0.088	77	2.74	75.33	−0.1866	−0.1676	−0.0270	0.0483	0.1406
43	29.7	39.94	−0.135	0.124	−0.094	95	0.94	79.57	−0.1680	−0.1559	−0.0196	0.0578	0.1363
44	22.9	33.98	−0.172	0.092	−0.124	103	2.12	108.71	−0.1469	−0.1396	0.0198	0.0740	0.1594
45	13.6	23.49	−0.154	0.097	−0.123	95	1.11	106.81	−0.1575	−0.1199	0.0079	0.0598	0.1278
46	19.2	31.23	−0.177	0.091	−0.129	101	2.09	117.95	−0.1441	−0.1339	0.0239	0.0242	0.1578
47	27.4	38.24	−0.146	0.116	−0.105	87	1.15	102.06	−0.1671	−0.1541	−0.0077	0.0492	0.1464
48	28.3	36.95	−0.145	0.119	−0.102	155	0.43	128.38	−0.1674	−0.1497	−0.0160	0.0373	0.1337
49	29.7	38.29	−0.144	0.110	−0.104	99	1.54	100.34	−0.1652	−0.1425	−0.0049	0.0734	0.1377
50	13.2	12.49	−0.207	0.038	−0.121	121	2.63	132.08	−0.1339	−0.1263	−0.0011	0.0267	0.1252

Table 2. *Cont.*

No. [b,e]	Expt. [c]	ΔH_{homo} [d]	Q_Y	Q_N	Q_O	N_X	μ	α	E_{Homo-1}	E_{Homo}	E_{Lumo}	E_{Lumo+1}	ΔE
51	12.4	11.85	−0.203	0.035	−0.124	137	3.28	153.74	−0.1234	−0.1153	0.0033	0.0337	0.1186
52	13.1	11.72	−0.208	0.041	−0.118	137	4.84	143.34	−0.1427	−0.1348	−0.0109	0.0158	0.1240
53	14.5	12.36	−0.207	0.040	−0.118	143	3.15	158.34	−0.1387	−0.1307	−0.0080	0.0150	0.1227
54	32.5	48.21	−0.146	0.125	−0.094	79	3.83	77.38	−0.1599	−0.1557	−0.0060	0.0435	0.1497
55	32.8	48.03	−0.145	0.128	−0.090	71	2.96	68.62	−0.1787	−0.1652	−0.0112	0.0363	0.1541
56	33.9	48.00	−0.144	0.129	−0.089	63	2.45	60.05	−0.1814	−0.1680	−0.0138	0.0333	0.1542
57	34.3	48.06	−0.145	0.130	−0.088	97	1.58	72.48	−0.1799	−0.1709	−0.0164	0.0273	0.1545
58	38.6	47.95	−0.144	0.135	−0.081	85	1.04	74.82	−0.1663	−0.1594	−0.0271	-0.0070	0.1323
59	35.0	47.76	−0.147	0.128	−0.088	31	1.92	24.78	−0.1833	−0.1690	−0.0136	0.0815	0.1554
60	37.9	47.74	−0.148	0.126	−0.089	39	1.99	30.96	−0.1813	−0.1665	−0.0118	0.0848	0.1547
61	36.7	47.60	−0.149	0.126	−0.090	47	2.06	36.87	−0.1797	−0.1641	−0.0108	0.0857	0.1533
62	33.7	52.20	−0.110	0.100	−0.133	73	3.07	69.25	−0.1633	−0.1419	0.0196	0.0800	0.1615
63	33.7	51.64	−0.109	0.102	−0.131	65	2.37	62.62	−0.1929	−0.1580	0.0166	0.0717	0.1746
64	35.0	51.41	−0.109	0.103	−0.131	57	2.07	54.70	−0.1987	−0.1606	0.0147	0.0687	0.1753
65	36.2	51.26	−0.109	0.104	−0.129	91	1.51	65.59	−0.1785	−0.1607	0.0117	0.0596	0.1724
66	36.2	50.62	−0.108	0.108	−0.126	79	2.29	69.83	−0.1609	−0.1539	0.0036	0.0037	0.1575
67	21.0	40.30	0.191	−0.013	−0.102	73	1.54	73.16	−0.1346	−0.1224	0.0074	0.0892	0.1298
68	21.4	42.55	0.194	−0.010	−0.098	65	1.22	66.29	−0.1429	−0.1356	0.0027	0.0878	0.1384
69	19.4	42.36	0.195	−0.009	−0.096	57	0.93	57.96	−0.1469	−0.1385	0.0003	0.0853	0.1388
70	19.2	41.90	0.203	−0.005	−0.089	73	2.16	66.58	−0.1566	−0.1503	−0.0116	0.0655	0.1388
71	18.6	41.20	0.207	−0.002	−0.085	79	3.17	75.23	−0.1538	−0.1466	−0.0180	0.0149	0.1286
72	23.4	43.03	0.170	−0.024	−0.107	145	1.49	140.97	−0.1496	−0.1263	0.0129	0.0746	0.1393
73	20.9	41.43	0.204	−0.003	−0.087	73	2.79	65.26	−0.1594	−0.1513	−0.0121	0.0654	0.1392
74	19.3	38.80	0.186	0.015	−0.088	73	2.72	63.76	−0.1609	−0.1405	−0.0022	0.0525	0.1384
75	19.9	42.46	0.194	−0.010	−0.098	65	0.94	65.19	−0.1444	−0.1363	0.0023	0.0874	0.1386
76	25.0	46.38	0.151	−0.034	−0.114	49	1.80	39.73	−0.1443	−0.1181	0.0220	0.1478	0.1401
77	17.2	39.26	0.179	0.002	−0.103	73	1.83	69.31	−0.1394	−0.1197	0.0138	0.0779	0.1334
78	24.4	45.63	0.149	−0.027	−0.117	179	2.30	140.93	−0.1304	−0.1189	0.0212	0.0634	0.1402
79	24.3	44.57	0.159	−0.044	−0.110	161	4.67	112.48	−0.1408	−0.1370	0.0018	0.0560	0.1388
80	26.2	45.85	0.150	−0.029	−0.116	171	1.87	119.96	−0.1210	−0.1094	0.0193	0.0730	0.1287
81	26.1	47.32	0.184	−0.058	−0.099	131	4.00	95.00	−0.1422	−0.1267	−0.0061	0.0910	0.1206
82	26.6	47.09	0.193	−0.057	−0.093	163	3.82	117.85	−0.1487	−0.1157	−0.0129	0.0625	0.1028
83	29.2	45.93	0.148	−0.026	−0.117	163	2.20	116.77	−0.1297	−0.1175	0.0228	0.0752	0.1402
84	27.4	45.55	0.150	−0.028	−0.117	139	1.95	98.97	−0.1309	−0.1186	0.0217	0.0741	0.1402
85	28.8	31.13	0.051	0.039	−0.090	79	1.75	71.97	−0.1562	−0.1397	−0.0079	0.0321	0.1318
86	29.2	35.79	0.023	0.035	−0.090	85	1.87	77.42	−0.1568	−0.1368	−0.0059	0.0292	0.1309
87	27.5	32.66	−0.026	0.050	−0.080	117	2.33	83.36	−0.1571	−0.1373	−0.0043	0.0782	0.1330
88	27.6	30.10	−0.023	0.041	−0.082	109	0.77	78.48	−0.1560	−0.1312	0.0004	0.0811	0.1316
89	26.2	37.46	0.144	0.027	−0.068	69	3.01	51.18	−0.1491	−0.1416	−0.0172	0.0286	0.1245
90	30.4	31.02	−0.019	0.042	−0.086	101	2.00	72.83	−0.1542	−0.1313	0.0021	0.0808	0.1334
91	31.4	37.38	0.162	0.034	−0.067	47	2.49	34.34	−0.1558	−0.1485	−0.0200	0.0222	0.1285
92	26.3	27.64	−0.013	0.037	−0.081	61	1.79	49.55	−0.1531	−0.1414	−0.0114	0.0481	0.1300

[a] Unit: ΔH_{homo} (kcal mol^{-1}), Charge (e), Dipole Moment (debye), Polarizability (a.u.) and Energy (a.u.); [b] 1~53 contain N-NO, 54~66 contain O-NO, 67~84 contain S-NO, 85~92 contain C-NO; [c] Measured in CH3CN at 25 ℃ by titration calorimetry; [d] The calculated homolysis bond dissociation energies are with zero-point energy (ZPE) and thermal corrections to enthalpy at 298K by B3LYP/STO-3G.
